# Risk factors for postoperative pancreatic fistula after laparoscopic distal pancreatectomy using stapler closure technique from one single surgeon

**DOI:** 10.1371/journal.pone.0172857

**Published:** 2017-02-24

**Authors:** Tao Xia, Jia-Yu Zhou, Yi-Ping Mou, Xiao-Wu Xu, Ren-Chao Zhang, Yu-Cheng Zhou, Rong-Gao Chen, Chao Lu, Chao-Jie Huang

**Affiliations:** 1 Department of Surgery, Zhejiang University School of Medicine, Hangzhou, Zhejiang Province, China; 2 Department of Gastrointestinal-Pancreatic Surgery, Zhejiang Provincial Peoples’ Hospital, Hangzhou, Zhejiang Province, China; Virginia Mason Medical Center, UNITED STATES

## Abstract

Laparoscopic distal pancreatectomy (LDP) is a safe and reliable treatment for tumors in the body and tail of the pancreas. Postoperative pancreatic fistula (POPF) is a common complication of pancreatic surgery. Despite improvement in mortality, the rate of POPF still remains high and unsolved. To identify risk factors for POPF after laparoscopic distal pancreatectomy, clinicopathological variables on 120 patients who underwent LDP with stapler closure were retrospectively analyzed. Univariate and multivariate analyses were performed to identify risk factors for POPF. The rate of overall and clinically significant POPF was 30.8% and13.3%, respectively. Higher BMI (≥25kg/m^2^) (p-value = 0.025) and longer operative time (p-value = 0.021) were associated with overall POPF but not clinically significant POPF. Soft parenchymal texture was significantly associated with both overall (p-value = 0.012) and clinically significant POPF (p-value = 0.000). In multivariable analyses, parenchymal texture (OR, 2.933, P-value = 0.011) and operative time (OR, 1.008, P-value = 0.022) were risk factors for overall POPF. Parenchymal texture was an independent predictive factor for clinically significant POPF (OR, 7.400, P-value = 0.001).

## Introduction

Distal pancreatectomy (DP) is the standard procedure performed for benign or malignant lesions in the body or tail of the pancreas. Mortality from these procedures has decreased considerably however the rate of complications has still remained high. Recently minimally invasive approach to performing distal pancreatectomy has been become more common. Compared to open distal pancreatectomy (OPD), patients undergoing laparoscopic distal pancreatectomy (LDP) are reported to have lower blood loss, fewer postoperative complications and a shorter length of stay (LOS) without a substantial increase in the operative time [1–3]. Despite these benefits, the rate of complications still remains high and varies between 30–40%, the most common being postoperative pancreatic fistula (POPF), wound infections and omental infarcts [4]. POPF is the major source of postoperative morbidity and is associated with numerous further complications, such as intra-abdominal abscess, sepsis, and hemorrhage [5]. The texture of the gland, duct diameter, technique of resection and closure of the pancreatic remnant and experience of surgeon have all been identified as risk factors. Although many ways have been described to prevent POPF, no consensus on avoiding POPF has yet been defined.

Although some studies show that stapler closure and hand-sewn closure have no differences in POPF [6–8], stapler closure is considered to be safe and approved [9–11]. At our institution stump closure was performed using the staple technique. The objective of this study was to analyze the risk factors for POPF after LDP.

## Subjects and methods

### Ethics statement

Approval for this investigation was obtained from the ethical committees of Zhejiang Provincial Peoples’ Hospital. The obtaining informed consent was waived because of a retrospective study without collecting personal identities.

### Study design

This investigation was a retrospective case-control study of patients underwent LDP with stapler closure of the pancreatic remnant.

### Study subjects

The inclusion criteria were patients: 1) who were aged more than 18 years; 2) who had undergone LDP. Between March 2011 and March 2016, Clinicopathological variables of 120 patients were collected retrospectively on the general demographics, intraoperative and pathological findings and postoperative outcomes including gender, age, body mass index (BMI), surgical technique, estimated blood loss (EBL), operative time, pathology, pancreatic fistula, and time to oral intake after operation.

### Standard treatment protocols

All patients underwent contrast-enhanced abdominal computed tomography (CT) or abdominal magnetic resonance imaging (MRI) as part of their preoperative evaluation. All patients received prophylactic antibiotics preoperatively. Somatostatin analogue was given according to drain amylase. Abdominal CT was also obtained at 3 to 7 days after the operation to assess any postoperative morbidity. Drain tube was removed depending on the volume, drain and serum amylase levels.

### Surgical technique

All surgeries were performed by one single surgeon. Five trocars were inserted, including a camera port (10mm) below the umbilicus and four additional working ports (one 12mm and three 5mm) which were placed in the right flank, right upper flank, left upper flank, left flank. Carbon dioxide pneumoperitoneum was maintained at 12mmHg -15mmHg.

The gastrocolic ligament was divided to expose the anterior surface of the pancreas. Dissection was performed in a medial-to-lateral fashion to expose the superior and inferior pancreatic margins. The splenic vessels were then identified and dissected free from the posterior surface of the pancreas. Once adequate mobilization of the pancreas was achieved, the linear stapler (white load, 2.5mm) was applied to transect the pancreatic parenchymal. The slow parenchymal flattening technique was used when transecting the pancreas[12]. Endoscopic linear cutter was mechanical (ETHICON ENDO-SURGERY, EC60A) prior to January 2014, and after January 2014, the power-driven cutter was used (ETHICON ENDO-SURGERY, PSE60A). Although it was a subjective way of assessment, a soft or hard pancreas was determined by the surgeon’s tactile response of the instrument and reconfirmed after being taken out from the peritoneal cavity. Dissection was continued to the splenic hilum along the pancreas and splenic vessels. The short gastric vessels were reserved carefully. Kimura and Warshaw techniques were used for preserving the spleen[13, 14]. If appropriate vascular inflow and outflow through spleen was not preserved and the spleen was ischemic, splenectomy was performed which was also for oncologic principle. The surgical specimen was removed through a minimal incision using a specimen bag. A drainage tube was placed beside the stump of pancreas but not too close and extracted from the right abdominal wall.

### Definitions

POPF was defined according to the International Study Group on Pancreatic Fistula Definition (ISGPF) as a drain output of on or after postoperative day 3 with an amylase value greater than 3 times the serum amylase [15]. Three different grades of POPF (Grades A, B, C) were defined according to the patient’s hospital course. All patients with Grade B and C were defined as clinically significant POPF.

All patients with BMI≥25 kg/m^2^ were defined as being overweight according to World Health Organization(WHO) definition [16].

### Statistical analysis

All data were summarized as mean and standard deviation for continuous variables or median (interquartile range) for categorical variables frequency. Univariable analyses were performed using a Student’s t test for continuous variables. Fisher’s exact or Pearson’s chi-square test was used to compare categorical variables as appropriate. All variables with p<0.05 were tested in the multivariate analysis. Binary Logistic regressions were performed for multivariable analyses of parameters potentially associated with POPF. The odds ratio (OR) with a 95% confidence interval was reported. All statistical analyses were performed using SPSS v.21.0 for Microsoft Windows (IBM) statistical software package. All p-values < 0.05 were considered to be statistically significant.

## Results

One hundred and twenty patients underwent LDP. The median age of the patients was 50 (21–79) years and a majority were females (N = 76, 63.3%). The median BMI was 22 (16–32) kg/m^2^. The median tumor size was 4.7 (1.0–15.0) cm. The tumor was benign in 73 (60.8%) patients, 18 (15%) had were malignant potential disease, and 29 (24.2%) lesions were frankly malignant. Median operative time and estimate blood loss were 177 (75–445) minutes and 85 (10–520) mL respectively. 43 (35.8%) patients had fluid accumulation in the peritoneal cavity after CT evaluation and hydrops of 27 cases disappeared within three weeks. The drainage tube was removed in the hospital if no POPF. However, most patients were sent home with drains if length of stay was longer than 10 days with fistula. The median length of tube taking in Grade A fistula was 12 (6–18) days. Somatostatin analogue was used for 14 patients because the drain amylase was still high after three weeks. The median length of postoperative hospitalization was 9 (4–25) days, and 9 (7.5%) patients were readmitted to the hospital. Two (1.7%) underwent reoperation because of intraperitoneal hemorrhage, and one (0.8%) for deep vein thrombosis. Six (5%) patients had peripancreatic fluid collection, four (3.3%) of which were underwent percutaneous drainage. There was no postoperative mortality. Splenectomy was performed in 60 (50.0%) of the cases. For spleen preservation, 49 (81.7%) underwent the Kimura procedure, and 11 (18.3%) underwent the Warshaw procedure. Additional resections included hepatic wedge biopsy (N = 3, 2.5%), hepatic lobectomy (N = 1, 0.8%), cholecystectomy (N = 6, 5%), adrenalectomy (N = 1, 0.8%), and partial gastric resection (N = 4, 3.3%).

POPF was the most frequent complication, presenting in 38 (30.8%) patients. 21 (17.5%) were transient fistula (Grade A) and had no clinical impact. Sixteen (13.3%) patients had clinically significant POPF (Grade B, C), and of these four (3.3%) required percutaneous drainage. Higher BMI (≥25kg/m^2^) was associated with overall POPF (p-value = 0.025) but not clinically significant POPF (p-value = 0.281). Operative time was longer in all patients who experienced overall POPF (168.01±54.84 vs. 194.14±72.60, p-value = 0.021) but not those who experienced clinically significant POPF (p-value = 0.450). Soft parenchymal texture was significantly associated with both overall (p-value = 0.012) and clinically significant POPF (p-value = 0.000). There were no differences in sex, age, EBL, tumor size, time to oral intake, pancreatitis, pathology, and splenic preservation both in the overall and clinically significant POPF (Tables 1 and 2).

**Table 1:** Univariate analysis of risk factors for overall POPF after LDP (n = 120).

	No fistula	Grade A, B,C	P
Sex (male/female)			0.816
Male	31 (37.3%)	13 (35.1%)	
Female	52 (62.7%)	24 (64.9%)	
Age(years)	50.43±15.84	50.49±16.45	0.987
BMI(kg/m^2^)			0.025
<25	74 (89.2%)	27 (73%)	
≥25	9 (10.8%)	10 (27%)	
Operative time (min)	168.01±54.84	194.14±72.60	0.021
Estimate blood loss (mL)	80.24±71.55	97.03±90.30	0.277
Tumor size(cm)	4.90±2.82	4.28±2.37	0.249
Time to oral intake	3.13±1.97	2.95±1.60	0.613
Pathology			0.396
Benign	48 (57.8%)	25 (67.7%)	
Malignant potential	12 (14.5%)	6 (16.2%)	
Malignant	23 (27.7%)	6 (16.2%)	
Splenic preservation			0.323
Yes	39(47%)	21 (56.8%)	
No	44(53%)	16 (43.2%)	
Pancreatitis			1.000
Yes	4(4.8%)	2(5.4%)	
No	79(95.2%)	35(94.6%)	
Pancreatic texture			0.012
Soft	23(27.7%)	19(51.4%)	
Hard	60(72.3%)	18 (48.6%)	

**Table 2:** Univariate analysis of risk factors for clinically significant POPF after LDP (n = 120).

	No fistula/ Grade A	Grade B, C	P
Sex			0.629
Male	39 (37.5%)	5 (31.3%)	
Female	65 (62.5%)	11 (68.8%)	
Age(years)	50.62±16.05	49.38±15.85	0.774
BMI(kg/m^2^)			0.281
<25	89 (85.6%)	12 (14.4%)	
≥25	12 (75%)	4 (25%)	
Operative time (minutes)	175.00±62.26	187.63±60.65	0.450
Estimate blood loss (ml)	82.98±71.24	101.25±113.48	0.384
Tumor size(cm)	4.70±2.80	4.88±1.98	0.806
Time to oral intake	3.13±1.90	2.69±1.54	0.372
Pathology			0.496
Benign	62 (59.6%)	11(68.8%)	
Malignant potential	15 (14.4%)	3 (18.8%)	
Malignant	27 (26%)	2 (12.5%)	
Splenic preservation			1.000
Yes	52(50%)	8 (50%)	
No	52(50%)	8 (50%)	
Pancreatitis			1.000
Yes	5(4.8)	1(6.3%)	
No	99(95.2)	15(93.8)	
Pancreatic texture			0.000
Soft	30(28.8%)	12(75%)	
Hard	74(71.2%)	4 (25%)	

BMI, operative time and parenchymal texture were further examined in a multivariate analysis. Parenchymal texture (OR, 2.933, p-value = 0.011) and operative time (OR, 1.008, p-value = 0.022) were risk factors for overall POPF (Table 3). The other multivariate analysis was also performed with potential variables including pancreatic texture, BMI, operative time, and pathology. Parenchymal texture was an independent predictive factor for clinically significant POPF (OR, 7.400, P-value = 0.001) (Table 4).

**Table 3:** Multivariate analysis of risk factors for overall POPF.

Variables	OR	95%CI	P
Operative time (min)	1.008	1.001–1.104	0.022
BMI(kg/m^2^)	1.961	0.664–5.789	0.223
Pancreatic texture	2.933	1.281–6.713	0.011

**Table 4:** Multivariate analysis of risk factors for clinically significant POPF.

Variable	OR	95%CI	P
Pancreatic texture	7.400	2.210–24.778	0.001
BMI(kg/m^2^)	0.977	0.230–4.149	0.975
Operative time(min)	1.007	0.997–1.106	0.160
Pathology	0.490	0.223–1.074	0.075

## Discussion

Laparoscopic distal pancreatectomy has more advantages than open surgery, such as lower blood loss, fewer postoperative complications and a shorter length of stay (LOS). However, there is no difference in POPF between LPD and OPD [1, 17, 18]. A systematic review [19] demonstrated that POPF rates varied from 3.7% to 68.5% in distal pancreatectomy using different criteria. ISGPF developed a consensus definition and grading scale to aid in classifying POPF according to the clinical severity [15]. POPF rates using ISGPF in the present study was 30.8%. Grade A fistula was 17.5%, which requires little or no change in the clinical management of the patient. The incidence of clinically significant POPF (Grade B, C) was 13.3%. POPF rates don’t deviate significantly from DISPACT trial (stapler 32% vs. hand sewn 28%) [7] and other LDP data [20–22].

From a surgical point of view, POPF in particular is an unsolved issue. There have been reported several potential factors influence the occurrence of POPF, including patient-related risk factors (age, sex, and BMI), disease-related risk factors (pancreatic gland texture and pancreatic duct size), procedure-related risk factors (operative time, transection technique, closure technique, and intraoperative blood loss), and the surgeon’s experience. But until now the superiority of any methods has not been demonstrated convincingly.

Several non-randomized and randomized studies aim for a surgical solution of this situation. The most favored and reported techniques are stapler closure vs. hand-sewn closure of the pancreatic remnant. It also includes laparoscopic approaches, radiofrequency-assisted dissection procedure, and biological glue [22, 23]. Systematic reviews [6, 8] and randomized study [7] showed that there were no significant differences between stapler closure and hand-sewn closure in POPF. But stapler closure is considered to be simple and safe [9–11]. So the pancreatic remnant stump closure was performed using the staple technique in this study. Although the endoscopic linear cutter was mechanical and power-driven, there was no significant difference between power-driven and mechanical linear cutter. To exclude the influence of surgeon’s experience, all patients were performed by one single surgeon using surgical standardization. White loan staplers (2.5mm) were used in 80.5% patients in this retrospective data, although it may be too small for pancreas[24]. Lager staplers (3.5–4.5mm) and suture reinforcement were also used if the pancreas was too thick. To exclude the effect of different staplers, only white loan staplers were included in this study.

In our study, splenectomy and spleen preservation were carried out in 50% (n = 60) of the cases, respectively. Spleen preservation was not a risk factor for POPF. The impact of splenectomy and splenic preservation on fistula development remains controversial. There are no significant differences between splenic preservation and splenectomy influenced POPF in previous studies [25–27]. However, a meta-analysis showed that the rate of POPF defined in any way was not significantly different between splenectomy and splenic preservation (OR, 0.87, p-value = 0.58) with moderate heterogeneity (I^2^ = 37%). Nonetheless the rate of clinically significant POPF was significantly lower in the splenic preservation group (6.90 vs.14.33%; p-value = 0.002) with low heterogeneity (I^2^ = 0%) [28]. A report indicated that factors significantly associated with POPF were male sex and spleen-preservation [29].This is because potential devascularization of the pancreatic remnant in splenic preservation caused the wound-healing process in the pancreatic stump to fail. Furthermore, it has been proved that blood supply at the cut surface of the pancreas is an important factor for pancreatic fistula after pancreaticoduodenectomy [30]. Conversely, Ridolfino, et al [31] including 64 patients showed splenectomy was associated with a clinically-relevant fistula, which was confirmed by Goh, et al with analysis of 232 patients [32].

In OPD, Kleeff, et al reported an operating time≥480 minutes was associated with POPF [25]. An analysis identified operative time≥300 minutes as the only notable predictor of clinically relevant POPF (OR = 3.253) [33]. In this study operative time was a risk factor for overall POPF but not clinically significant POPF. From literatures, longer operative time is related with higher BMI, fatty visceral fat area [34, 35], It is also related with multivisceral resections and malignant neoplasm radical operation [31].

In this Study, BMI≥25 was a risk factor for overall POPF. Weber, et al reported BMI>27 was a factor for major fistula formation after LPD [36]. Other studies showed an increase in intra-abdominal complications, pancreatic fistula, and mortality in patients with an increasing BMI (BMI>30 kg/m^2^, BMI>25 kg/m^2^) after OPD [26, 37, 38].

Few studies have reported pancreatic texture and pancreatitis as predictive factors for POPF after DP. Okano, et al, demonstrated that fibrotic pancreas was more likely to develop POPF when a linear stapler was used [12]. The stump of chronic pancreatitis is believed to be a lower POPF rate for holding sutures more securely. However chronic pancreatitis didn’t have a lower fistula rate than soft pancreas (28% vs. 29%)[26]. Even chronic pancreatitis is also a risk factor for POPF using hand-sewn closure [39]. This is due to downstream stenosis of the main pancreatic duct, most likely in the pancreatic head region. However chronic pancreatitis was not a risk factor for POPF in present study. Maybe it is due to few of chronic pancreatitis cases (5%) for illustrating. However hard parenchymal texture is not equal to pancreatitis texture. On the contrary, soft pancreas is a significant risk factor for POPF in previous studies [40, 41]. Soft pancreatic texture was also a significant risk factor for overall and clinically significant POPF using a linear stapler in this study. Unek T, et al, [41] proved that the soft pancreatic parenchyma texture was a risk factor for POPF (OR: 12.420, p-value = 0.048) and "U" shaped sutures was a method to reduce POPF. But Stump closure of soft texture is not uniform to prevent POPF after DP. Recently pancreatic thickness is concerned increasingly. Nakamura M, et al, showed high BMI value and thick pancreatic stump were significant risk factors for POPF after LDP [42]. Mendoza, et al [43], detected pancreatic parenchymal texture alone was not an independent risk factor but pancreatic thickness was a significant predictive factor for POPF, also the thick and soft combination was particularly significant for POPF. Using stapler closure, a thicker pancreatic stump is increased POPF after DP [44, 45]. Furthermore, a stapler closure seems to be suitable at least for thin pancreas [12, 46]. A drawback of this study is no thickness data, so the relationship between stapler, pancreatic texture and thickness is not entirely clear. From literature review, in our opinions, POPF is mainly associated with pancreatic texture, thickness and closure technique. There was no uniform guideline on which closure technique to according to pancreatic texture and thickness. It needs further randomized clinical trials.

There were some limitations of this study. Firstly, it was a retrospective analysis; hence we were unable to collect some significant data, which might affect the outcomes, such as diameter of pancreatic duct, thickness of the pancreatic stump. Secondly, postoperative outcomes were not collected elaborately.

## Conclusions

BMI≥25 was a risk factor for overall POPF. Operative time was a risk factor for overall POP. Soft pancreatic texture is the only significant risk factor for both overall and clinical POPF in this study. There may be some correlation between BMI, visceral fat, operation time, and soft pancreatic texture, but it should be proved through further study such as evaluating visceral fat by radiography.
